# Is it time to reconsider traditional medicine?: a commentary

**DOI:** 10.1177/17449871251347158

**Published:** 2025-10-29

**Authors:** Pernilla Garmy

**Affiliations:** Professor, Faculty of Health Sciences, Kristianstad University, Kristianstad, Sweden

Commentary to: A clinical trial of Arnebia euchroma ointment and silver nanoparticle dressings on pain relief and pressure ulcer healing (Sarhadi et al. (2025)).

In an era where modern pharmaceuticals can be both expensive and environmentally burdensome, and where side effects are a frequent concern, it is important not to overlook the value of plant-based knowledge – often locally available and passed down through generations. At the same time, we must not adopt traditional medicine uncritically. All forms of care and treatment, whether modern or traditional, deserve rigorous scientific evaluation. As we know, herbal medicine can be just as potent – and potentially harmful – as synthetic alternatives.

In the present randomised controlled trial by Sarhadi et al. (2025), the authors examine whether a traditional Persian herbal ointment made from *Arnebia euchroma* can stand up to modern wound care using silver nanoparticles dressings. Pressure ulcers are a major source of suffering and generate significant healthcare costs. They are associated with pain, long healing periods and intensive care needs. In countries such as Iran, advanced wound dressings are often not reimbursed by health insurance, highlighting the urgent need for affordable alternatives.

*Arnebia euchroma*, a plant native to southeastern Iran, has long been used in Persian traditional medicine. Its root contains bioactive compounds such as shikonin, alkannins and naphthoquinones – known for their wound-healing, antifungal, antiviral, antimicrobial, and anti-inflammatory properties. These compounds help reduce oxidative stress and inflammation while supporting tissue repair. The root also includes sterols, steroidal glycosides and triterpenes, which contribute analgesic effects without known toxic side effects (Fan et al., 2013).

Silver-based wound dressings are a common standard of care for pressure ulcers, yet silver can cause side effects and poses environmental concerns. This makes it especially relevant to investigate alternative or complementary approaches.

For ethical reasons, the trial compared *Arnebia euchroma* ointment to silver nanoparticle dressings rather than a placebo, as all patients required appropriate care. The study was conducted in two ICUs in Iran and included 60 patients – 30 in each treatment group.

Pain was assessed on days 1, 7 and 12 using the Non-Verbal Pain Scale, measured before, during and after dressing changes. Wound size was assessed using the Pressure Ulcer Scale for Healing (PUSH) at the same intervals.

The findings indicate that both treatments were effective in promoting wound-healing. However, patients in the *Arnebia euchroma* group experienced significantly less pain during and after dressing changes. While both groups showed a reduction in ulcer size, the difference between them was not statistically significant.

This is one of the first studies to compare *Arnebia euchroma* ointment with silver nanoparticle dressings for the treatment of pressure ulcers, focusing on both healing and pain management. Given that the long-term effects of silver nanoparticles on human health and the environment are still uncertain, *Arnebia euchroma* emerges as a promising alternative – either as a standalone therapy or in combination with silver-based treatments.
